# Causality in dietary interventions—building a case for gut microbiota

**DOI:** 10.1186/s13073-018-0573-y

**Published:** 2018-08-01

**Authors:** Yan Y. Lam, Chenhong Zhang, Liping Zhao

**Affiliations:** 1 Department of Biochemistry and Microbiology and New Jersey Institute for Food, Nutrition, and Health, School of Environmental and Biological Sciences, Rutgers University, NJ, 08901 USA; 2 State Key Laboratory of Microbial Metabolism and Ministry of Education Key Laboratory of Systems Biomedicine, School of Life Sciences and Biotechnology, Shanghai Jiao Tong University, Shanghai, 200240 China

## Abstract

We provide a conceptual framework to establish a causal link between diet, gut microbiota, and health. Identifying the key strains that mediate microbe–host interactions and understanding the mechanisms involved and the ecology of these strains are critical to translating gut microbiome research into clinical applications and to advancing a new concept of “microbiome nutrition”.

## Causality is the missing link in our understanding of the gut microbiome, nutrition, and health

The gut microbiome has been at the forefront of medical research for the past decade, and many expect that these microorganisms will soon become a key part of health management. The biggest hurdle in translating gut microbiome research into clinical applications is demonstrating the causative role of the gut microbiome in human health and diseases. It is now clear that diet is very powerful in changing the gut microbiota (for better or worse) and studies of dietary effects may be an important approach for understanding causality in regard to the gut microbiome. Here, we provide a conceptual framework, guided by a series of questions, to demonstrate how the causative role of the gut microbiome in nutrition and health can be elucidated and to advance a new concept of “microbiome nutrition”.

### Does the gut microbiota play a causative role?

Proof of causality starts with correlative evidence. The key here is a sophisticated experimental design that explicitly implicates the gut microbiome as a causative agent of dietary effects. Studies of prebiotics or dietary fibers are relatively straightforward as these typically comprise non-digestible but fermentable carbohydrates that work exclusively on the gut microbiota, so they are the sole contributor to any observed effects. Randomized controlled trials in which added fermentable carbohydrates are the only dependent variable would imply a causative role for the gut microbiome in any measured clinical benefits [1]. Such causality may not, however, be that obvious for most dietary interventions that modulate both the gut microbiota and other host pathways. Consider diet-induced weight loss—the gut microbiota changes when we lose weight [2], but does this change occur as a cause or as a consequence of weight loss, caloric restriction, and/or change in macronutrient ratios? Transplantation of gut microbiota into germ-free mice, for example, is one of the best existing models with which to demonstrate causality in such cases. In our recent study involving patients with type 2 diabetes, mice receiving post-intervention gut microbiota from fiber-supplemented individuals exhibited improved glucose homeostasis that mirrored the clinical effects of high fiber intake [1]. Applying a similar concept, fecal microbiota transplantation from severely stunted or underweight children to germ-free mice induced significant weight loss in the animals and this established a causal relationship between the gut microbiota and the undernourished phenotype [3]. Here, the combination of clinical trials and a gnotobiotic animal model (fecal microbiota transplantation) demonstrates that the gut microbiota is at least partly responsible for the clinical outcomes.

### Which are the key strains?

Causality at the level of the whole-gut microbiota will need to be narrowed down to key players at the strain level. Conventional taxonomy-based analysis overlooks strain-specific functionality, as strains within the same species can have up to 30% difference in their genomic makeup. The assembly of high-quality draft genomes from shotgun metagenomic sequencing of longitudinal samples from dietary interventions allows strain-level analysis of the predominant members of the human gut microbiota. Two extreme and yet complementary approaches are necessary to dissect the diet-modified gut ecosystem and to identify the specific strains that are of interest.

First, we need to go big and to study the gut microbiota as a system using an ecological perspective. The microbiota that make up the gut ecosystem do not exist in isolation but they interact and work with each other. We explored the interrelationships between (or the network of) predominant bacterial strains when the gut ecosystem was perturbed by a large amount of non-digestible but fermentable carbohydrates and observed distinct patterns of co-abundant changes. Bacterial strains that co-occur (they thrive or decline together) can be considered as a “guild” in ecology. Members of a guild respond to a stimulus (for example, a change in resource availability) in a similar way and most probably also provide similar function(s) or service(s) to the human host. Applying this concept, we identified a guild of 15 short-chain fatty acid producers that was selectively promoted by dietary fibers [1]. When bacteria were clustered on the basis of how they responded to dietary interventions, the guild-level abundance showed strong correlations with host parameters, and some guilds were even able to predict clinical improvements [1, 4]. Guilds with the strongest correlations to host parameters warrant further investigation.

Now we need to go small and to isolate members of these guilds either into pure cultures or as a consortium with defined membership. Such cultured strains can be used to establish gnotobiotic models of the disease, that is, to reproduce disease phenotype(s) in animals by introducing cultures of a single bacterial strain or a defined consortium into the gut. We established a gnotobiotic model of obesity by colonizing germ-free mice with a pure culture of *Enterobacter cloacae* B29, a strain that overgrew in the gut of a morbidly obese individual [5]. Similarly, Surana and Kasper [6] showed that *Clostridium immunis*, a single *Lachnospiraceae* isolate from the feces of mice colonized with human gut microbiota, conferred protection against experimentally induced colitis, which is consistent with the clinical observation of an inverse relationship between *Lachnospiraceae* abundance and the risk of inflammatory bowel disease. We can also use this strategy to demonstrate the protective effects of beneficial strains. For example, the pure culture of *Bifidobacterium pseudocatenulatum* C95, a member of the guild of short-chain fatty acid producers identified in patients with type 2 diabetes, mitigated deleterious metabolic sequelae in diet-induced obese mice and ameliorated hyperglycemia in germ-free mice colonized with baseline gut microbiota from these patients [1]. Such a strategy demonstrated the causative role of these strains in metabolic health following the logic of Koch’s postulates.

### What do the key strains do?

Next, we need to characterize the functions of the strains of interest and to elucidate the mechanisms by which they interact with host tissues. A key to understanding molecular mechanisms is to use a combination of in vitro culture, animal models, and clinical trials that offers insights into biochemistry as well as clinical relevance. A notable example is the work on the “leaky gut” and metabolic endotoxemia theory. This started with seminal work from Cani and colleagues that identified the impairment of a gut barrier as a key mechanism by which microbial metabolites harbored by diet-induced obese mice, specifically lipopolysaccharides, trigger systemic inflammation [7].

Extension of this work then focused on why the gut becomes leaky. Data from Caco-2 culture and *Akkermansia muciniphila*-inoculated mice showed that Toll-like receptors and the endocannabinoid system are the key signaling components through which microbial metabolites and membrane proteins interact with the host to regulate the paracellular permeability of the gut epithelium [8, 9]. Identifying these molecular targets in the host may present opportunities to block or enhance specific microbial effects without actually manipulating the gut microbiota, which could have profound consequences. Recently, host parameters, for example, hyperglycemia [10], have also been shown to play a role in regulating gut barrier integrity, suggesting that certain phenotypes may make the host more susceptible to microbe-induced effects. However, these mechanistic studies used animals or humans with the existing gut microbiota and did not identify key members that drive the molecular changes. Examining the effects of isolated metabolites, pure cultures, or cultures of defined membership in vitro and in gnotobiotic animals are necessary approaches to understanding the biochemistry of microbe–host interactions.

### How do we understand the key strains in ecology?

A final question that we need to consider is why some gut microbes, among trillions of their counterparts, are able to become predominant and make significant contributions to the microbial impact on the host. Here we need to take a step back and understand the ecology of the gut microbiota. What makes some microbes work together and form a guild? What is the relationship between guilds? Are some guilds more important than others? The microbiome field has long advocated for the application of ecological concepts so that we can understand the gut microbiota as an ecosystem [11], but only recently, with our study as an example, has microbial ecology been used to interpret dietary effects. Our data suggest that the availability of resources, for example, energy substrates from the host diet, creates a selective pressure on members of the microbial community; dominance of certain groups of microbes may then modify the environment that then impacts on other members. We showed that dietary fibers induced the bloom of a guild of acetate and/or butyrate producers, which lowered gut pH and inhibited metabolically detrimental bacteria that are less tolerant of an acidic environment [1]. This ties in with another important concept in ecology—some members (guilds) may be “foundation species”. For example, just as tall trees with overlapping crowns can form a closed canopy and prevent sunlight from reaching other plants in a forest, foundation species change the environment and can therefore shift the structure of the microbiota, and this shift can be beneficial for human health. Accordingly, understanding the dynamics of the gut ecosystem and identifying the key drivers of microbial ecology would be crucial for developing preventative and therapeutic strategies that target the gut microbiota.

## Microbiome nutrition: The new way of healthy eating

Deciphering causality is pivotal to putting gut microbiota into the clinical setting. We have discussed how to use a combination of clinical, animal, and in vitro studies to systematically identify and to understand the key gut microbes. This information is crucial to our ability to harness the potential of gut microbiota to improve our health. We see diet as the foundation of gut microbiome-targeted interventions—it effectively shifts the microbial structure, and most importantly, it can create an environment that promotes the long-term stability of a healthy microbiota. Dietary programs that are specifically designed to modify the gut microbiota have already been shown to benefit weight management and glycemic control [1, 4], and similar benefits may also be available in other diseases. Collaborative efforts from microbiology, food science, and nutrition are needed to re-invent the way that we eat—emphasizing the need to “feed me and my microbes” and putting “microbiome nutrition” at the center of novel therapeutic strategies to treat and prevent diseases.
